# Evaluating freshwater macroinvertebrates from eDNA metabarcoding: A river Nalón case study

**DOI:** 10.1371/journal.pone.0201741

**Published:** 2018-08-08

**Authors:** Sara Fernández, Saúl Rodríguez, Jose L. Martínez, Yaisel J. Borrell, Alba Ardura, Eva García-Vázquez

**Affiliations:** 1 Department of Functional Biology, University of Oviedo, Oviedo, Asturias, Spain; 2 Unit of DNA Analysis, Scientific-Technical Services, University of Oviedo, Oviedo, Asturias, Spain; Oklahoma State University, UNITED STATES

## Abstract

Rivers are a vital resource for human wellbeing. To reduce human impact on water bodies, the European Union has established an essential regulatory framework for protection and sustainable management (WFD; 2000/60/EC). In this strategy, reliable and economic bioindicators are a fundamental component. Benthic macroinvertebrates are the group most commonly used as bioindicators through all European countries. However, their conventional assessment currently entails serious cost-efficiency limitations. In this study, we have tested the reliability of metabarcoding as a tool to record river macroinvertebrates using samples from a mock community (*in vitro* validation) and eDNA extracted for field validation from water from six sites within a north Iberian river (River Nalón, Asturias, Spain). Two markers (V4 region within the nuclear 18S rDNA and a fragment of the mitochondrial COI gene) were amplified and sequenced using an Illumina platform. The molecular technique has proven to be more sensitive than the visual one. A cost-benefit analysis shows that the metabarcoding approach is more expensive than conventional techniques for determining macroinvertebrate communities but requires fewer sampling and identification efforts. Our results suggest metabarcoding is a useful tool for alternative assessment of freshwater quality.

## Introduction

Rivers are one of the most important resources for human society, supplying the population with different goods and services: from drinking and industrial water to fisheries to recreational activities [1]. Due to these anthropogenic uses, running water ecosystems are constantly changing and have generally experienced a reduction in the ecosystem services they provide [2]. As an attempt to reduce the impacts on European water bodies, the European Water Framework Directive (WFD; 2000/60/EC) has established a framework for their protection and sustainable management, with the aim of achieving at least a `good water status’ [3]. Good water quality is one of the essential requirements to accomplish the status required within this directive.

Multiple indicator groups (macrobenthic fauna, fish fauna, and aquatic flora) have been widely used to measure the ecological quality of rivers across Europe [4–8]. Benthic macroinvertebrates are biotic indicators of water quality because they reflect a diversity of anthropogenic perturbations, thus serving to detect both habitat and overall stream degradation [9]. They are organisms that usually inhabit the bottom substrates and are large enough to be seen without magnification. The dominant groups are arthropods, mollusks, and annelids [10]. Their use as bioindicators is widespread across Europe, and, together with algae, they are the most common biological water quality assessment indicators [9]. For these reasons, the monitoring of resident macroinvertebrate communities has become a primary component of water-resource evaluations with regard to the WFD [11].

Collection and identification of macroinvertebrates with traditional methodologies is generally costly. It requires a high sampling effort and the contribution of expert taxonomists for morphological identification that is sometimes difficult to obtain because of the lack of diagnostic characteristics for many macrozoobenthic larvae [12].

However, the use of environmental DNA (eDNA), where the genetic material is obtained directly from environmental samples (soil, sediment, water, etc.) [13], could overcome these cost-efficiency limitations. The samples needed for applying eDNA-based methodologies are easy to collect without the need for sampling individuals from the river, which can be difficult in river zones with no accessibility to the river bottom or in areas where netting is inefficient because of a low or nonexistent current. Due to the substantial number of taxa that compose ‘benthic macroinvertebrates’, from arthropods to annelids, the use of a metabarcoding approach appears to be a good option. Metabarcoding has been defined as the combination of high-throughput sequencing (HTS) platforms and DNA sequence association with taxonomic information to surveying [14]. Although it requires next-generation sequencing (NGS) technologies and the use of expensive platforms, the process can be externalized to specialized companies, reducing costs and becoming relatively affordable for monitoring aquatic communities [15]. NGS has been used to assess macroinvertebrates in a few studies [16–19], demonstrating its potential ability to monitor such a varied group of organisms. Within the mentioned studies, some authors have used a metabarcoding approach to assess benthic macroinvertebrates from tissue samples [19,20], showing its feasibility and higher sensitivity than morphological methods. Others validated the use of NGS for environmental samples to evaluate water quality in marine ecosystems [16] and in biodiversity studies in freshwater ecosystems [17], including macroinvertebrate species assessment. The application of these technologies to environmental samples is increasing [21]. Most of the recently developed studies have been based on advancing eDNA based approaches implementation (e.g., [13,21,22,23]), focusing on field validation, platform and barcode choice or database limitations [24–26]. However, there is a lack of information about the reliability of taxonomic assignment criteria. In this study, we tested the reliability of next-generation sequencing (NGS) for the detection and identification of macroinvertebrate families from running water samples using two different metabarcodes for checking the consistency of the taxonomic assignments and determining the proportion of positive and negative results by comparison of eDNA results with physical macroinvertebrate samples from the field, and a mock community created *in vitro* from known DNA samples. Field samples obtained along a river will also serve to test the hypothesis of rivers being like conveyer belts of biodiversity [17]. From this hypothesis, DNA from terrestrial species will be found in water samples as well, so the assessment using eDNA could cover landscapes. And it is expected that the species diversity will increase downstream for macroinvertebrates and for the whole community identified from eDNA.

## Methods

### Ethics statement

This project, and in particular the collection of samples in protected spaces, was authorized by the entity legally entitled to do so in Spain, the Government of the Asturias Principality, with permit reference 101/16. The authors adhered to the European Code of Conduct for Research Integrity (ESF 2011).

### Sample collection

**Fig 1:**
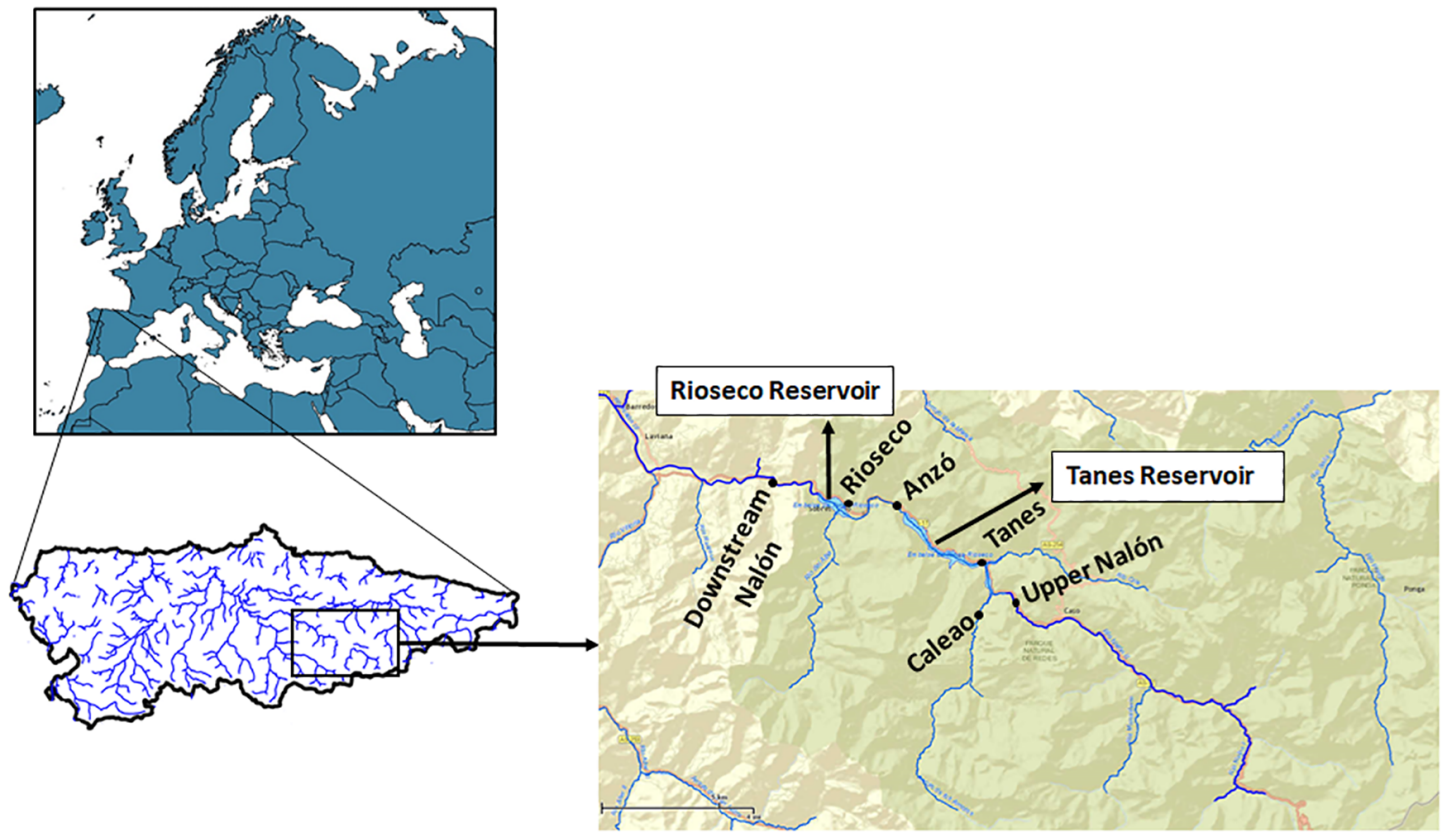
Upper Nalón basin. Distribution of sampling points along the Upper Nalón River. The two reservoirs in the area are indicated (Rioseco and Tanes reservoirs).

Water samples were collected in November 2016 from six sites along the upper zone of the Nalón River (Fig 1), a river area belonging to the Nalón-Narcea basin in Asturias in the north of Spain. Study sites are located within the UNESCO (United Nations Educational, Scientific and Cultural Organization) Biosphere Reserve and the Redes Natural Park, a protected area with high faunal diversity [27]. In this area, river connectivity is interrupted by the presence of two big dams (Fig 1).

At each site, four liters of water were collected with a sterile bottle placed at the river bottom without disturbing the sediment. One liter of Milli-Q water was transported to the field and analyzed in the laboratory with the rest of the samples to monitor for contamination. After water sample collection, macroinvertebrate individuals were sampled after superficially kicking the riverbed substrates about for one minute (Kick-net method), as is performed in conventional macroinvertebrate sampling [28]. The released individuals were then collected with a 0.09 m^2^ stainless steel sieve (1-μm mesh). The specimens collected were identified down to the family level using an identification key [29].

### Processing and next-generation sequencing

Four one-liter samples were analyzed per sampling point. These water samples and the Milli-Q negative control were vacuum filtered using a Supor^®^ 200 Membrane Filter (Pall Corporation, Life Sciences, Ann Arbor, MI, USA) with 0.2-μm pore size. The filtration room was free of external sources of contamination, and it was separate from the molecular laboratory. The filtration system was cleaned with 10% commercial chlorine-based bleach between samples to avoid contamination between sampling points. Milli-Q water was filtered as the last sample, following the same steps to monitor for filtration cross-contamination. Lastly, the filters were placed into 15 mL tubes using sterile forceps and stored at −20 °C until DNA extraction.

DNA was extracted from filters with the PowerWater^®^ DNA Isolation Kit (QIAGEN laboratories) under sterile conditions inside a laminar flow PCR-cabinet following the manufacturer’s instructions. A negative control was added at this step to monitor contamination during the extraction process.

Metabarcoding molecular work was performed at the Cawthron Institute (www.cawthron.org.nz). PCR was performed for two target genes, the eukaryotic V4 region of the nuclear small subunit ribosomal DNA (18S rRNA gene, 18S from now) using the universal primers Uni18SF and Uni18SR [30] and a mitochondrial COI gene region using the universal primers COI NexF-mlCOIintF and NexR-jgHCO2198 [31]. The primers were modified to include Illumina^™^ overhang adaptors.

PCR for the 18S gene was performed on an Eppendorf Mastercycler (Eppendorf, Germany) in a total volume of 35 μl containing 18 μl of AmpliTaq Gold^®^ 360 PCR Master Mix (Life Technologies, USA), 5 μl of AmpliTaq PCR Enhancer (Life Technologies, USA), 2 μl of BSA, 1 μM of each primer, and 3 μl of template DNA. The reaction cycling conditions were as follows: 95 °C for 3 min; followed by 35 cycles of 94 °C for 30 s, 52 °C for 30 s, and 72 °C for 90 s; and a final extension at 72 °C for 8 min. PCR of the COI gene was performed in a total volume of 35 μl containing 1x MyTaq^™^Red Mix (Bioline, USA), 1 μM of each primer and 3 μl of template DNA. The reaction cycling conditions were as follows: 95 °C for 1 min; followed by 35 cycles of 95 °C for 15 s, 46 °C for 15 s, and 72 °C for 10 s; and a final extension at 72 °C for 3 min. Negative and positive controls were included for all PCR reactions. The amplification success was visually assessed on a 1.5% agarose gel.

PCR amplicons were purified using the AMPureTM XP system (Agenecourt, USA), quantified using the QuBit BR dsDNA kit (Invitrogen, USA), diluted to a concentration of 3 ng/μl and sent to New Zealand Genomics Limited (University of Auckland) for library preparation and sequencing. Sequencing adaptors and sample-specific indices were added to each amplicon via a second round of PCR using the Nextera^™^ Index kit (Illumina^™^) following the manufacturer’s instructions. Amplicons were pooled into a single library and paired-end sequences (2 × 250) were generated on a MiSeq instrument using the TruSeq^™^ SBS kit v3 (Illumina^™^). The MiSeq Control Software Version 2.2 including MiSeq Reporter 2.2 was used for raw read primary analysis and demultiplexing and to assign the forward and reverse reads to the samples.

### Bioinformatics analyses

Run quality was assessed using three processes, SolexaQA++, fastQC and fastQscreen. Using the VSEARCH tool [32], the pair-end reads from each sample were merged, filtered (discarding all reads with >1 error per assembled read and reads that were too long and too short compared to the expected amplicon length) and dereplicated into unique sequences. Chimeras were identified and removed in *de novo* mode using the UCHIME algorithm [33]. All the sequence reads were assessed for quality by applying a Phred quality score threshold of 30 (Table 1; Cleaned). Then, BLAST alignment was completed for the 18S rDNA dataset (maximum E-value = 10^−50^ and minimum percent identity = 80.0) against NCBI 18S sequences using QIIME [34].

**Table 1:** HTS and pipeline output. The number of sequences obtained along the process in the six samples analyzed and the Mock community for each gene. The sequences remaining after bioinformatics filtering (Merged and Cleaned) and the following different assignment criteria: #1 (maximum E-value = 10^−10^ and minimum percent identity = 97.0); #2 (maximum E-value = 10^−50^ and minimum percent identity = 97.0); #3 (maximum E-value = 10^−10^ and minimum percent identity = 90.0); #4 (maximum E-value = 10^−50^ and minimum percent identity = 90.0); and 18S Assigned (maximum E-value = 10^−50^ and minimum percent identity = 80.0).

	18S				COI						
Sample	Raw	Merged	Cleaned	Assigned	Raw	Merged	Cleaned	Assigned			
								Criteria#1	Criteria#2	Criteria#3	Criteria#4
C	91464	58671	32413	29253	126701	116138	113204	32507	5218	94383	15889
N	127708	85499	49037	45067	137023	127237	124397	29890	7550	108993	22052
T	95941	64785	53671	43050	199512	158686	154766	31224	1956	117332	12327
EE	114814	77896	44074	38823	254680	228560	222835	108083	16100	187941	32368
R	56441	32407	24433	23191	56074	52845	51497	29517	25076	70618	34884
NB	112483	88051	70862	64364	149794	139942	136701	39918	8269	114634	16589
Mock community	30604	10490	8739	8728	34132	32468	31781	628	4613	31746	31593
% of Assigned OTU				89%				32%	8%	87%	20%

For COI, BLAST alignment was also performed against NCBI COI sequences using QIIME, but with four different threshold criteria to further determine the most adequate for macroinvertebrate family assignation: Criteria #1 (maximum E-value = 10^−10^ and minimum percent identity = 97.0); Criteria #2 (maximum E-value = 10^−50^ and minimum percent identity = 97.0); Criteria #3 (maximum E-value = 10^−10^ and minimum percent identity = 90.0); and Criteria #4 (maximum E-value = 10^−50^ and minimum percent identity = 90.0). The E-value or Expect value is the number of different alignments with scores equivalent to or better than S (the raw alignment score), which is expected to occur in a database search by chance. The lower the E-value, the more significant the score and the alignment. The percentage of identity measures the extent to which two sequences have the same nucleotides at the same positions in an alignment [35]. The two partial NCBI databases (for 18S and COI genes) were built using the algorithm described by Baker [36] in 2017. Genetic assignments for both markers were performed by employing the ‘‘assign_taxonomy. py” python script. Reference databases were constructed using the work flow developed by Baker [36]. Finally, OTU (Operational Taxonomic Unit) tables, a list of OTUs obtained for each sample and the number of sequences assigned to them, were constructed with the ‘fromTaxassignments2OtuMap.py’ algorithm.

### *In vitro* and field validation

#### *In vitro* validation

A mock community was set up to verify that our laboratory methods and bioinformatics pipeline were able to correctly detect the taxa of interest (Table 2). It was composed of a known DNA mixture of nine species from different taxonomic groups (one crustacean, one insect, two acorn barnacles, two goose barnacles, and three fish) that occur in water samples at any life stage. This mock community was analyzed together with the set of eDNA samples obtained from the field. The taxonomic assignation of raw sequences for the mock community was manually checked with the BLAST tool included on the NCBI webpage [35] to confirm the assignations were correctly done using our pipeline or if there were errors or incongruences.

**Table 2:** Mock community results for the COI and 18S genes. Assignation results after different assignment methods (1–4 and 18S) and manual blast checking using the BLAST tool on the NCBI webpage [39]. Filtering criteria: #1 (maximum E-value = 10^−10^ and minimum percent identity = 97.0); #2 (maximum E-value = 10^−50^ and minimum percent identity = 97.0); #3 (maximum E-value = 10^−10^ and minimum percent identity = 90.0); #4 (maximum E-value = 10^−50^ and minimum percent identity = 90.0); and 18S (maximum E-value = 10^−50^ and minimum percent identity = 80.0).

MOCK COMMUNITY			NGS					Manual BLAST								
Family	Species	Quantity (ng)	COI				18S	COI					18S			
			Criteria #1	Criteria #2	Criteria #3	Criteria #4		Best match species	Accession number	E-value	Identity (%)	N Seqs	Best match species	Accession number	E-value	identity (%)
Caprellidae (crustacean)	*Caprella andreae*	0.05	-	-	-	-	-	-	-	-	-	120	-	-	-	-
Heptageniidae (insect)	*Rhithrogena sp*.	5	0	0	26941	26464	7829	*Rhithrogena sp*.	HM481023	e-133	94	450	*Rhithrogena sp*.	DQ008182	e-102	89
Salmonidae (fish)	*Salmo trutta*	5	4005	4002	4011	4011	-	*Salmo trutta*	HM480831	e-174	98	289	-	-	-	-
Lepadidae (goose barnacle)	*Lepas anatifera*	0.5	449	447	449	449	3	*Lepas anatifera*	GU993620	e-164	98	47	*Lepas anatifera*	FJ906773	e-146	96
Salmonidae (fish)	*Oncorhynchus mykiss*	0.5	20	20	22	22	-	*Oncorhynchus mykiss*	KU867889	e-175	98	304	-	-	-	-
Salmonidae (fish)	*Salmo salar*	0.05	18	18	18	18	772	*Salmo salar*	HM480828	e-178	98	305	*Oncorhynchus mykiss and other species*.	KY115616	0	99
Lepadidae (goose barnacle)	*Lepas pectinata*	0.05	69	69	68	68	-	*Lepas pectinata*	GU993658	e-164	98	17	-	-	-	-
Chthamalidae (acorn barnacle)	*Chthamalus stellatus*	5	28	28	29	29	2	*Chthamalus stellatus*	EU699251	e-166	98	34	*Chthamalus dalli and other species*	KM974371	0	99
Austrobalanidae (acorn barnacle)	*Austrominius modestus*	0.5	14	14	22	22	-	*Austrominius modestus*	KT209230	e-162	98	19	-	-	-	-

#### Field validation and statistics

The field validation was based on the coincidences between families found from the direct individual sampling of macroinvertebrates—taxonomically classified *de visu*—and the families found from metabarcoding at the six sampling points.

Alpha diversity was estimated using species richness (S). This index was chosen as representative of simple indices that give greater weight to rare species and are better than compound indices for detecting diversity disturbances (e.g.[37]). The statistical significance of the differences between diversity indices from different sites was determined by employing permutation tests. For these tests, 9999 random matrices with two columns (samples) are generated, each with the same row and column totals as in the original data matrix.

To check if there were significant differences between the two different molecular markers and the visual methodology, the Fisher’s exact test (based on contingency tables) was employed.

The diversity indices and statistical tests were computed using the PAST software [38].

### Cost-benefit analysis (CBA)

A CBA was performed following the methodology explained in Borrell *et al*. [15]. Briefly, the time employed performing molecular and morphological analyses was calculated for each step to estimate an effort measurement (sampling, extraction and identification processes). The cost of both methods was calculated based on the Spanish official technician wage (10.83€/hour), as the study took place in Spain. Laboratory costs for DNA extraction included filters for retrieving DNA from water samples and the costs of DNA extraction kits. Sequencing costs charged from Cawthron Institute (where the samples were analyzed) were also added for the metabarcoding approach.

## Results

### High-throughput sequencing and pipeline output

Good quality 18S amplicons were obtained for all the analyzed samples, while good quality COI amplicons were obtained for 20 of 24 samples. Raw NGS sequences are available on NCBI’s sequence read archive (SRA) with the Study number SRP124881.

The number of raw sequences obtained varied from 91,464 to 127,708 sequences per sample for the 18S region and from 56,074 to 254,680 sequences per sample for the COI fragment (Table 1). Sequence quality filtering (cleaning) retained 45% of 18S regions and 87.2% of COI sequences. The percentage of assigned COI sequences ranged from 8% with Criteria#2 to 87% with Criteria#3. A total of 89% of 18S sequences were assigned with the criteria followed for this DNA region (Table 1).

### *In vitro* validation

#### COI gene

From the Mock community, 8 of the 9 species added were detected. One of the added species, the crustacean *Caprella andreae*, was not detected with any of the criteria employed. For manual BLAST (Table 2, right) all the sequences obtained from NGS were correctly assigned to at least the genus level with a 90% identity threshold. Using the 97% identity threshold (Criteria#1 and #2), *Rhithrogena sp*. could not be assigned to a species because this sequence has a maximum of 94% identity with the references available in the NCBI database (see manual BLAST).

The number of sequences assigned to the reference species in the mock community was not proportional to the DNA quantity for each species. Even though the same amount of extracted DNA was added for *Rhithrogena sp*., *Salmo trutta*, and *Chthamalus stellatus* (5 ng), there is an enormous difference in the number of assigned sequences, with only 28–29 sequences being assigned to *Chthamalus stellatus* compared with 26,941–26,464 and 4,002–4,011 sequences assigned to *Rhithrogena sp*. and *Salmo trutta*, respectively. Differences were also found in the rest of the species assignations. Even though the same amount of DNA (0.5 ng) was added for *Lepas anatifera*, *Oncorhynchus mykiss* and *Austrominius modestus*, the number of assigned sequences was much higher for *Lepas anatifera* than for the other two species (Table 2). For *Salmo salar* and *Lepas pectinata* species, the number of sequences assigned to the detected species were 18–19 and 69, respectively (Table 2). Finally, 0.05 ng of *Caprella andreae* DNA was not detected.

Regarding the assignment criteria tested here, only one was able to correctly detect eight of the species present in the mock community with no false positives, Criteria#4 (E-value of e10^-50^ and 97% identity thresholds; Table 2, left). Using an E-value of e10^-10^, false positives appeared for 97% (Criteria#1, 47 sequences were incorrectly assigned to the fish *Myctophum lychnobium*) and 90% (Criteria#3, 19 sequences wrongly assigned to the arachnid *Teutonia cometes*) identity thresholds. Regarding false negatives, the insect *Rhitrogena* sp. could not be detected from Criteria#1 or #2 (Table 2, left).

#### 18S gene

For the 18S gene, five species from the DNA added to the mock community were not assigned (*Caprella andreae*, *Salmo trutta*, *Oncorhynchus mykiss*, *Lepas pectinata*, and *Austrominius modestus)*. There were 12 sequences for one nematode species that were wrongly assigned (*Eumonhystera cf*. *hungarica)*, and two of the assignments were under low quality criteria (*Salmo salar* and *Chthamalus stellatus*). Low quality criteria refers to sequences that were aligned using the BLAST tool on the NCBI webpage [39] (Manual BLAST). The real added species (Query) had the same punctuation of assignment (score, identity and coverage) with various species (best match species) (Table 2), so it was not possible to determine the best match. For the 18S gene, the number of sequences assigned correctly to the reference species from the mock community were roughly proportional to the DNA quantity of each species, but we can only refer to *Rhithrogena sp*. and *Lepas anatifera*, as incongruences were not found.

### Field validation

**Fig 2:**
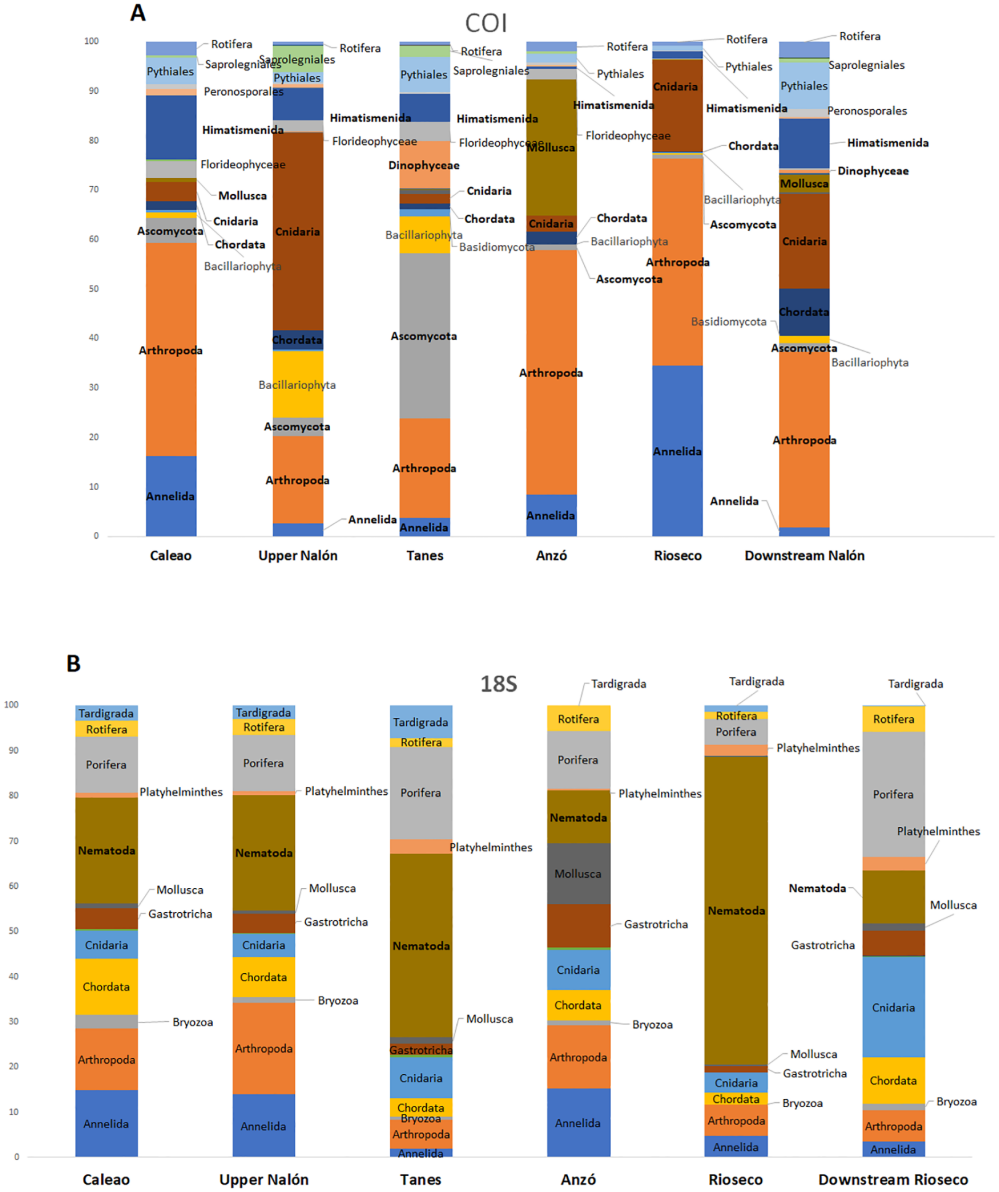
Taxonomic composition of the community identified from eDNA in the six sampling points considered from the Upper Nalón river. **A**: Percentage of sequences for each taxonomic group found per sampling point with the COI gene. **B**: Percentage of sequences for each taxonomic group found per sampling point with the 18S gene.

The overall taxonomic composition found in the analyzed sampling points was different depending on the genetic barcode employed (Fig 2).

More taxonomic groups were found with COI barcodes, which detected red algae, diatoms, and fungi; these organisms remained undetected with the 18S barcode. In decreasing order of abundance, the more relevant macroinvertebrate groups detected with the COI gene are as follows: Arthropoda > Cnidaria > Annelida > Mollusca. The order was different for the 18S barcode, as follows: Nematoda > Porifera > Arthropoda > Cnidaria (Fig 2). Many terrestrial species were found in the water from the two metabarcodes (S1 and S2 Tables), such as the birds *Cincla cincla* (European dipper) and *Passer domesticus* (sparrow) and many insects without an aquatic phase (Lepidoptera, etc.) that can be found on the river banks or nearby.

The community composition was different at the different sampling points. For example, the fungi Ascomycota were much more abundant at the Tanes sampling point for the COI marker than at the other points, while the abundance of Mollusca DNA was much higher at Anzó than at the other points (Fig 2 and S1 Table).

**Fig 3:**
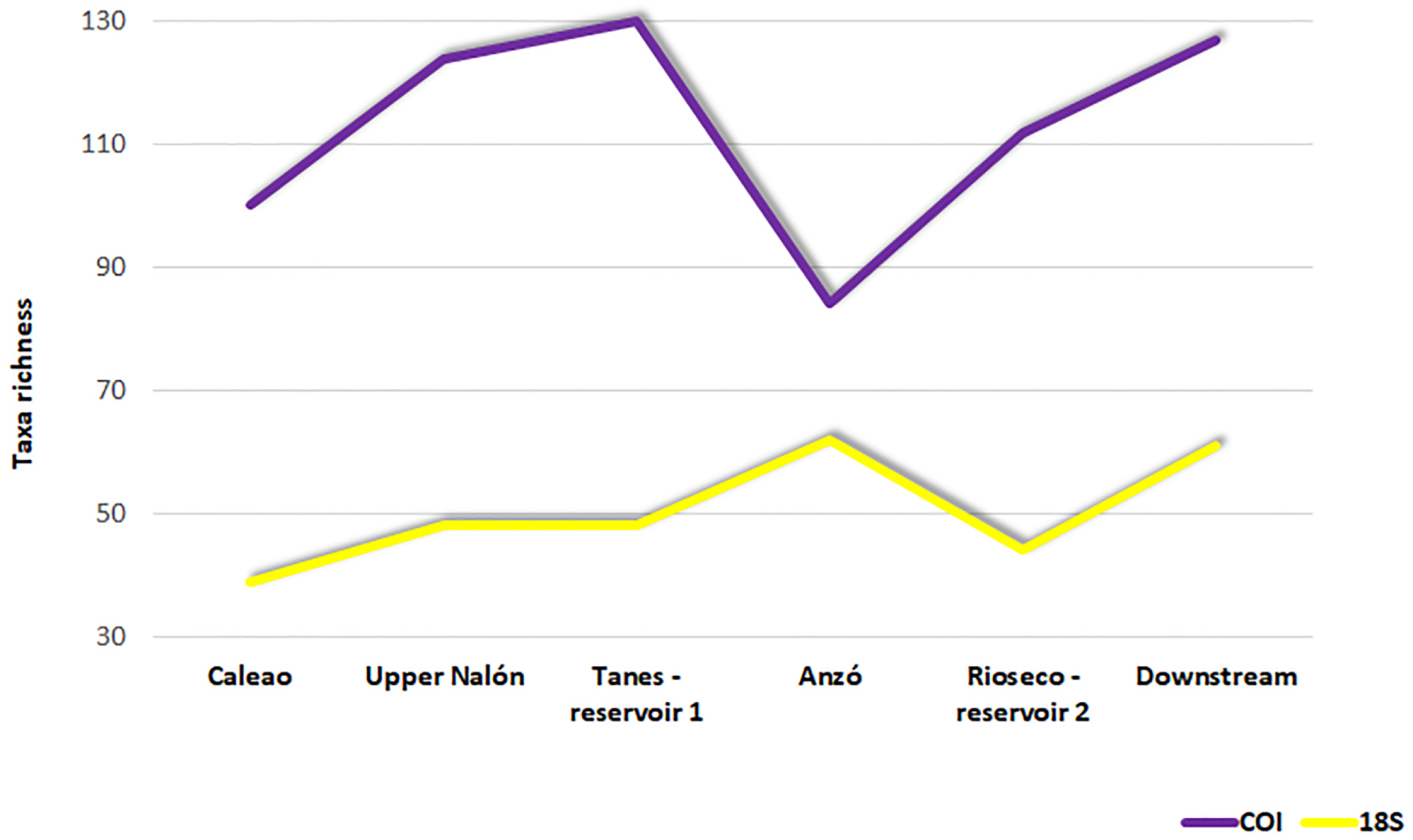
Genus richness at the six sampling points analyzed in this study within the Nalón river using COI and 18S metabarcodes. The points are ordered with downstream on the right.

Considering only freshwater Metazoans for a more homogenous biota profile when comparing the two barcodes and genus richness given the less accurate taxonomic identification of the 18S barcode, the taxa richness was different at the six sampling points using COI and 18S as barcodes (Fig 3).

The diversity decreased at one (18S barcode) or more (COI barcode) points within the area affected by reservoirs, with a minimum at Rioseco and Anzó in the respective datasets. For the COI marker, the decrease at Anzó was so sharp that this point was significantly different from the diversity at all the other points, except upstream at Caleao (Table 3).

**Table 3:** P-values obtained by permutations for pairwise differences in genus richness between the sampling points considered in the Nalón river. Significant values after Bonferroni correction are marked in bold.

COI/18S	Caleao	Upper Nalón	Tanes	Anzó	Rioseco	Downstream
Caleao	-	0.0173	0.2029	0.032	0.3623	0.0359
Upper Nalón	**0.0001**	-	1	0.0464	0.4309	0.185
Tanes	**0.0001**	0.0153	-	0.0506	0.3952	0.2086
Anzó	0.1003	**0.0013**	**0.0003**	-	0.0341	0.8509
Rioseco	0.1622	0.185	0.0855	**0.0001**	-	0.0569
Downstream	0.0127	0.5654	0.5715	**0.0001**	**0.0001**	-

For the 18S metabarcode (Table 3, above diagonal), no significant differences were found for any pairwise comparisons after applying Bonferroni correction (threshold of P = 0.0083 for significance). The point located downstream exhibited the highest diversity in the two datasets, but this was not significantly different from several points upstream for any metabarcode.

Regarding the macroinvertebrate indicators of water quality for the EU WFD, nineteen families were found by visual observation at the sampling points from the River Nalón basin (Table 4).

**Table 4:** Comparisons between methods. Macroinvertebrate families found by visual observation (visu) and through next-generation sequencing employing the 18S and COI genes, with Assignment criteria #4 for the latter, at each sampling point (marked with “X”). Proportion of false negatives considering all the sampling sites. Number of positives: the number of times each family was detected through sampling points with each methodology (COI,18S and visual); employed to calculate Fisher´s exact test.

	Caleao			Upper Nalón			Tanes			Anzó			Rioseco			Downstream Nalón			Number of positives		
Family	visu	COI	18S	visu	COI	18S	visu	COI	18S	visu	COI	18S	visu	COI	18S	visu	COI	18S	visu	COI	18S
Baetidae	X	X	X	X	X	X		X	X	X	X	X	X	X	X	X	X		5	6	5
Caenidae						X							X						1	0	1
Chironomidae		X	X	X	X	X		X	X	X	X	X	X	X	X		X	X	3	6	6
Chloroperlidae				X									X						2	0	0
Elmidae										X			X						2	0	0
Ephemerellidae	X	X	X		X	X			X			X			X	X		X	2	2	6
Heptageniidae	X	X	X	X	X	X					X	X		X			X		2	5	3
Hydropsychidae		X			X					X	X	X		X			X	X	1	5	2
Leptoceridae	X									X				X					2	1	0
Leuctridae	X	X		X	X									X					2	2	0
Lumbricidae	X																		1	0	0
Lymnaeidae						X				X	X	X							1	1	2
Phylopotamidae													X						1	0	0
Planorbidae		X			X			X					X	X	X			X	1	4	2
Polycentropodidae				X							X			X		X	X		2	3	0
Sericostomatidae	X	X			X								X						2	2	0
Simuliidae		X			X		X	X			X			X			X		1	6	0
Sphaeriidae																X			1	0	0
Tipulidae		X			X		X	X						X		X			2	4	0
**N**	**7**	**10**	**4**	**6**	**10**	**6**	**2**	**5**	**3**	**6**	**7**	**6**	**8**	**9**	**4**	**5**	**6**	**4**	**34**	**47**	**27**
**False negatives**	**-**	**2**	**3**	**-**	**2**	**3**	**-**	**0**	**2**	**-**	**2**	**2**	**-**	**5**	**5**	**-**	**3**	**4**			
**False negative proportion**	**COI = 29.8%**																				
	**18S = 70.4%**																				

The same or a higher number of families than those detected by visual identification were found from each sampling point by employing the COI gene as the barcode (Table 4). Using the 18S gene, fewer families were found than with COI and from conventional sampling. The consistency between eDNA-based family detection and visual observation was higher for COI than for the 18S gene (56.25% and 20.59%, respectively). Considering all the sites, the differences in the number of positives for each family detected from the three methods were statistically significant (Chi-square of contingency value of 44.515 for 19 rows and 3 columns, Fisher’s exact test with P-value = 0.009). The 18S barcode was able to detect only 8 of the 19 families sampled from the river using the conventional methodology, while the COI barcode detected 13 of them. The Chloroperlidae, Elmidae, Lumbricidae, Phylopotamidae, and Sphaeriidae families remained undetected by the eDNA methodology (Table 4).

For false negatives, as expected from the previous results, the number of families found by visual observation at each site that were not detected by the metabarcoding approach was indeed higher for 18S than for the COI gene. However, the significant difference was only marginally (p<0.1) significant (Chi-square of 19.927 for 14 rows and 2 columns, Fisher’s exact test with P = 0.097, Monte Carlo P = 0.072).

### CBA results

The metabarcoding approach required less effort for sampling and identification (in time) than the morphological approach for sampling and sample processing (53 and 250 min, respectively) (Table 5).

**Table 5:** CBA. Cost estimates for effort and measurements for the metabarcoding and morphological approaches in Spain, where the study took place. Currency: euros (€).

*Effort per sample*	Metabarcoding	Morphological
Sampling in the river (time)	3 min	10 min
DNA extraction (time)	30 min	-
DNA extraction products (€)	12.7	-
Individual identification (time)	-	240 min
Sequencing cost (€)	40.58	-
Bioinformatics (time)	10 min	-
**Total time**	43 min	250 min
**Total cost**	61.04	45.12

The time estimated for bioinformatics assumes that only one criteria (Criteria#4 as determined in this study) is used; thus, it includes the time necessary for writing commands and retrieving the OTU table in the pipeline employed here. The whole price for the metabarcoding analyses was 61.04 euros per sample, which is higher than that estimated for the morphological approach in the current study. The CBA was calculated considering the number of minutes employed, the real metabarcoding costs, and the salaries of technicians in Spain.

## Discussion

Although uses of eDNA-based tools are continuously increasing [21,40–42], the molecular techniques employed, such as the metabarcoding approach, need to be validated depending on the research purposes. It is important to consider the choice of platform, barcode, and threshold criteria for bioinformatics analyses before the application of those procedures in real-life cases. In this study, we tested partial COI and 18S genes, two common barcodes for NGS analysis [20,43,44], and a combination of different assignation criteria. Here, we have been able to demonstrate the higher accuracy of the COI gene by employing exigent criteria, such as an E-value (10^−50^) and 90% identity. All the species were correctly assigned in the mock community, and assignment incongruences were not observed (Table 2). Although higher identity is generally employed for species assignation in normal barcoding using this gene [45,46], it should be considered that the taxonomic level analyzed for water quality indices is family [9,47], not species, and 90% appears to be enough to assign invertebrate sequences to the family level [42]. Using a more restrictive identity threshold (97%), we would lose some information [48], such as in the case of *Rhithrogena sp*. from the mock community (Table 2). In the mock community, the number of sequences assigned to a species was not proportional to the amount of DNA for that species. This could be explained from primer biases: some primers anneal preferentially to DNA from some taxonomic groups, a bias that has been reported by different authors [24,25]. In other cases, the lack of assignment could be explained from the few reference sequences in the current databases. This problem of reference scarcity has been repeatedly reported in many studies [21,49–51]. Expanding databases with barcodes from different regions, especially for underrepresented species, should be a priority for enabling the application of metabarcoding methodologies in real life environmental analysis.

The nuclear 18S gene did not provide reliable results in this study, and the reasons may be varied. After the quality filtering processes, a high proportion of COI sequences were left for assignation (87.2%; 835,181 sequences), while assignation of the 18S gene was only possible for 44% of the raw sequences (283,229 sequences). Despite the assignation criteria for the 18S gene being quite permissive (minimum percent identity = 80.0), 5 of the 9 species in the mock community could not be assigned (false negatives). Two of the nonassigned species, *Lepas pectinata* and *Austrominius modestus*, have 2 and 3 18S gene sequences, respectively, in the database; thus, they were probably not assigned because of the lack of reference sequences in the NCBI database. However, the same explanation does not fit for the lack of assignation for *Salmo trutta* and *Oncorhynchus mykiss*, as 18S gene sequences for these two species are more abundant in the database (211 and 495 sequences, respectively). Moreover, incongruent assignations were found for *Salmo salar* and *Chthamalus stellatus* in the mock community using the 18S gene (Table 2), with higher identity thresholds for various species. The results derived from the mock community showed that the 18S gene is not an appropriate barcode for metabarcoding analyses for our purpose. Additionally, the number of taxonomic groups assigned using the 18S marker was lower than the number assigned with the COI gene. A higher number of Arthropods were assigned with the COI marker; thus, for our purpose of identifying benthic macroinvertebrates that are mostly arthropods, the COI gene marker has been shown to be more appropriate.

The field results supported the choice of the COI fragment as the metabarcode for macroinvertebrate assessment, as it had a relatively low proportion of false negatives, at least in comparison with 18S (29.8% for the COI gene and 70.4% for the 18S gene).

In contrast, in the field results, though significant differences were not found between the markers and techniques (molecular or visual), more families were obtained from COI metabarcoding than from *de visu* analysis. Thus, the genetic techniques are generally more sensitive than conventional sampling [52,53]. It is possible that some invertebrates escaped manual sampling, especially if they were scarce or very small. Alternatively, it is possible that some floating DNA molecules were released from macroinvertebrates upstream. Another possibility that cannot be ruled out is that DNA is being released from carcasses or dead individuals deposited in the substrate. In any case, the presence of a species’ DNA indicated the species were or had been present at or near the sampling point.

The taxonomic composition of the sampled river points also contained terrestrial species (i.e., arachnids belonging to the arthropod group) (S1 Table), confirming the hypothesis that river eDNA incorporates biodiversity for a larger scale or whole landscapes [17]. However, in our study, the reservoirs interrupted the expected progressive increase in downstream diversity. Strong diversity decreases were observed in the zones with reservoirs; these results were more acute for COI than for the 18S metabarcode dataset. The differences between the two datasets can be explained by two factors. First, the COI metabarcode detected more genera than the 18S metabarcode; thus, greater statistical significance was obtained in pairwise comparisons. Second, some taxa more represented in the COI dataset, such as Mollusca and Annelida, do not have terrestrial life stages. Thus, they move into the water and their connectivity is interrupted by dams, while other taxa, like insects (more represented in the 18S dataset), can fly over the dam or pass it from the river’s edge in their adult phase. This suggests that the interruption of river connectivity, which is considered one of the worst ecological effects of dams and reservoirs [54–57], will differentially affect aquatic organisms depending on their life history.

From a more practical perspective, CBA estimation suggested that the conventional technique for macroinvertebrate assessment is costlier than the metabarcoding approach in effort, but not in monetary terms (metabarcoding approach is 15.92 euros more expensive than the conventional approach). Similar costs have been suggested by other authors [58,59], and the technical improvements and wider uses of metabarcoding will likely make the sequencing costs to go down. The use of an eDNA-based tool would therefore improve the effectivity and efficiency of water body assessment, allowing for the routine evaluation of freshwater ecosystems.

Finally, the results obtained in the present study regarding metabarcodes and taxonomic assignation criteria will lead the way for using metabarcoding in water samples as an alternative or complementary method for freshwater quality evaluation. As macroinvertebrates are most commonly used as bioindicators, standardizing this approach [13] will allow for increased efficiency and time management [43].

## Supporting information

S1 Table COI OTU Table. Raw data obtained with COI marker clustered in family OTUs (Operational Taxonomic Units). N_genus: Number of genus per family within sampling points. NA: non-asignment at that level.
(XLSX)

S2 Table 18S OTU Table. Raw data obtained with 18S marker clustered in family OTUs (Operational Taxonomic Units). N_genus: Number of genus per family within sampling points. NA: non-asignment at that level.
(XLSX)

## References

[1] Arthington AH, Naiman RJ, McClain ME, Nilsson C. Preserving the biodiversity and ecological services of rivers: New challenges and research opportunities. Freshw Biol. 2010;55: 1–16. 10.1111/j.1365-2427.2009.02340.x

[2] Malmqvist B, Rundle S. Threats to the running water ecosystems of the world. Environ Conserv. 2002;29: 134–153. 10.1017/S0376892902000097

[3] Muxika I, Borja Á, Bald J. Using historical data, expert judgement and multivariate analysis in assessing reference conditions and benthic ecological status, according to the European Water Framework Directive. Mar Pollut Bull. 2007;55: 16–29. 10.1016/j.marpolbul.2006.05.025 16844146

[4] Gabriels W, Lock K, De Pauw N, Goethals PLM. Multimetric Macroinvertebrate Index Flanders (MMIF) for biological assessment of rivers and lakes in Flanders (Belgium). Limnologica. Elsevier; 2010;40: 199–207. 10.1016/j.limno.2009.10.001

[5] Mondy CP, Villeneuve B, Archaimbault V, Usseglio-Polatera P. A new macroinvertebrate-based multimetric index (I 2M 2) to evaluate ecological quality of French wadeable streams fulfilling the WFD demands: A taxonomical and trait approach. Ecol Indic. 2012;18: 452–467. 10.1016/j.ecolind.2011.12.013

[6] Murphy JF, Davy-Bowker J, McFarland B, Ormerod SJ. A diagnostic biotic index for assessing acidity in sensitive streams in Britain. Ecol Indic. Elsevier Ltd; 2013;24: 562–572. 10.1016/j.ecolind.2012.08.014

[7] Stream D, Index F, Environmental D, Agency P, Danish T, Fauna S, et al. Stream assessment in Denmark: the Danish Stream Fauna Index (DSFI). Stream assessment in Denmark: the Danish Stream Fauna Index (DSFI) Introduction. 2003.

[8] Birk S, Bonne W, Borja A, Brucet S, Courrat A, Poikane S, et al Three hundred ways to assess Europe’s surface waters: An almost complete overview of biological methods to implement the Water Framework Directive. Ecol Indic. Elsevier; 2012;18: 31–41. 10.1016/J.ECOLIND.2011.10.009

[9] AQEM Consortium. Manual for the application of the AQEM system. 2002; 202 http://www.aqem.de/ftp/aqem_manual.zip

[10] Alba J, Pardo I, Prat N, Pujante A. Metodología para el establecimiento el Estado Ecológico según la Directiva Marco del Agua. Protocolos de muestreo y análisis para invertebrados bentónicos. Magrama. 2005.

[11] von der Ohe PC, Prüss A, Schäfer RB, Liess M, de Deckere E, Brack W. Water quality indices across Europe-A comparison of the good ecological status of five river basins. J Environ Monit. 2007;9: 970–978. 10.1039/b704699p 17726558

[12] Birk S. Review of European assessment methods for rivers and streams using Benthic Invertebrates, Aquatic Flora, Fish and Hydromorphology. 2003.

[13] Thomsen PF, Willerslev E. Environmental DNA—An emerging tool in conservation for monitoring past and present biodiversity. Biol Conserv. 2015;183: 4–18. 10.1016/j.biocon.2014.11.019

[14] Deiner K, Bik HM, Mächler E, Mathew S, Lacoursière-Roussel A, Altermatt F, et al Environmental DNA metabarcoding: transforming how we survey animal and plant communities. Mol Ecol. 10.1111/mec.14350 28921802

[15] Borrell YJ, Miralles L, Do Huu H, Mohammed-Geba K, Garcia-Vazquez E. DNA in a bottle—Rapid metabarcoding survey for early alerts of invasive species in ports. PLoS One. 2017;12 10.1371/journal.pone.0183347 28873426 PMC5584753

[16] Lejzerowicz F, Esling P, Pillet L, Wilding TA, Black KD, Pawlowski J. High-throughput sequencing and morphology perform equally well for benthic monitoring of marine ecosystems. Nat Publ Gr. 2015; 10.1038/srep13932 26355099 PMC4564730

[17] Deiner K, Fronhofer EA, Mächler E, Walser JC, Altermatt F. Environmental DNA reveals that rivers are conveyer belts of biodiversity information. Nat Commun. Nature Publishing Group; 2016;7: 12544 10.1038/ncomms12544 27572523 PMC5013555

[18] Carew ME, Pettigrove VJ, Metzeling L, Hoffmann AA. Environmental monitoring using next generation sequencing: Rapid identification of macroinvertebrate bioindicator species. Front Zool. 2013;10: 45 10.1186/1742-9994-10-45 23919569 PMC3750358

[19] Elbrecht V, Vamos EE, Meissner K, Aroviita J, Leese F. Assessing strengths and weaknesses of DNA metabarcoding-based macroinvertebrate identification for routine stream monitoring. Yu D, editor. Methods Ecol Evol. 2017;8: 1265–1275. 10.1111/2041-210X.12789

[20] Carew ME, Pettigrove VJ, Metzeling L, Hoffmann A a. Environmental monitoring using next generation sequencing: Rapid identification of macroinvertebrate bioindicator species. Front Zool. Frontiers in Zoology; 2013;10: 45 10.1186/1742-9994-10-45 23919569 PMC3750358

[21] Zaiko A, Martinez JL, Ardura A, Clusa L, Borrell YJ, Samuiloviene A, et al Detecting nuisance species using NGST: Methodology shortcomings and possible application in ballast water monitoring. Mar Environ Res. Elsevier Ltd; 2015;112: 64–72. 10.1016/j.marenvres.2015.07.002 26174116

[22] Ji Y, Ashton L, Pedley SM, Edwards DP, Tang Y, Nakamura A, et al Reliable, verifiable and efficient monitoring of biodiversity via metabarcoding. Ecol Lett. 2013;16: 1245–1257. 10.1111/ele.12162 23910579

[23] Aylagas E, Borja N, Rodríguez-Ezpeleta N. Environmental Status Assessment Using DNA Metabarcoding: Towards a Genetics Based Marine Biotic Index (gAMBI). PLoS One. 2014;9 10.1371/journal.pone.0090529 24603433 PMC3946187

[24] Cowart DA, Pinheiro M, Mouchel O, Maguer M, Grall J, Miné J, et al Metabarcoding Is Powerful yet Still Blind: A Comparative Analysis of Morphological and Molecular Surveys of Seagrass Communities. 2015; 10.1371/journal.pone.0117562 25668035 PMC4323199

[25] Lim NKM, Tay YC, Srivathsan A, Tan JWT, Kwik JTB, Baloğlu B, et al Next-generation freshwater bioassessment: eDNA metabarcoding with a conserved metazoan primer reveals species-rich and reservoir-specific communities. R Soc Open Sci. The Royal Society; 2016;3: 160635 10.1098/rsos.160635 28018653 PMC5180151

[26] Shaw JL a, Clarke LJ, Wedderburn SD, Barnes TC, Weyrich LS, Cooper A. Comparison of environmental DNA metabarcoding and conventional fish survey methods in a river system. Biol Conserv. Elsevier Ltd; 2016;197: 131–138. 10.1016/j.biocon.2016.03.010

[27] García-Ramos, J.C., Jiménez-Sánchez, M., Piñuela, L., Domínguez Cuesta, M.J, López Fernández C. Patrimonio geológico en Asturias: la cuenca alt a del río Nalón y la Cost a de los Dinosaurios. 2006.

[28] Alba-Tercedor J, Sánchez-Ortega A. UN MÉTODO RÁPIDO Y SIMPLE PARA EVALUAR LA CALIDAD BIOLÓGICA DE LAS AGUAS CORRIENTES BASADO EN EL DE HELLAWELL (1978). Limnética. 1978;4: 51–56. Available: http://www.limnetica.com/documentos/limnetica/limnetica-4-1-p-51.pdf

[29] Tachet, H., Bournaud, M., & Richoux P (1987). Introduction à l’étude des macroinvertébrés des eaux douces(systématique élémentaire et aperçu écologique). 1987.

[30] Zhan A, Hulák M, Sylvester F, Huang X, Adebayo AA, Abbott CL, et al High sensitivity of 454 pyrosequencing for detection of rare species in aquatic communities. Methods Ecol Evol. 2013;4: 558–565. 10.1111/2041-210X.12037

[31] Leray M, Yang JY, Meyer CP, Mills SC, Agudelo N, Ranwez V, et al A new versatile primer set targeting a short fragment of the mitochondrial COI region for metabarcoding metazoan diversity: application for characterizing coral reef fish gut contents. Front Zool. Frontiers in Zoology; 2013;10: 34 10.1186/1742-9994-10-34 23767809 PMC3686579

[32] Rognes T, Flouri T, Nichols B, Quince C, Mahé F. VSEARCH: a versatile open source tool for metagenomics. PeerJ. PeerJ Inc.; 2016;4: e2584 10.7717/peerj.2584 27781170 PMC5075697

[33] Edgar RC, Haas BJ, Clemente JC, Quince C, Knight R. UCHIME improves sensitivity and speed of chimera detection. Bioinformatics. Cambridge University Press, Cambridge, UK; 2011;27: 2194–2200. 10.1093/bioinformatics/btr381 21700674 PMC3150044

[34] Caporaso JG, Kuczynski J, Stombaugh J, Bittinger K, Bushman FD, Costello EK, et al QIIME allows analysis of high-throughput community sequencing data. Nat Methods. 2011;7: 335–336. 10.1038/nmeth.f.303 PMC3156573 20383131

[35] Altschul SF, Gish W, Miller W, Myers EW, Lipman DJ. Basic Local Alignment Search Tool. J Mol Biol. 1990;215: 403–410. 10.1016/S0022-2836(05)80360-2 2231712

[36] Baker C. Workflow for generating a qiime-compatible blast database from an entrez search.: 1–4.

[37] Morris EK, Caruso T, Buscot F, Fischer M, Hancock C, Maier TS, et al Choosing and using diversity indices: insights for ecological applications from the German Biodiversity Exploratories. Ecol Evol. Wiley-Blackwell; 2014;4: 3514–3524. 10.1002/ece3.1155 25478144 PMC4224527

[38] Hammer, Ø, Harper, D.A.T, Ryan & PD. PAST.Paleontological statistics software package for education and data analysis. 2001. p. 9.

[39] Altschul SF, Gish WR, Lipman DJ, Miller W, Myers EW. Basic local alignment search tool. J Mol Biol. 1990;215: 403–410. 10.1016/S0022-2836(05)80360-2 2231712

[40] Clusa L, Ardura A, Fernández S, Roca AA, García-Vázquez E. An extremely sensitive nested PCR-RFLP mitochondrial marker for detection and identification of salmonids in eDNA from water samples. PeerJ. 2017;5: e3045 10.7717/peerj.3045 28265514 PMC5333537

[41] Ardura A, Zaiko A, Martinez JL, Samulioviene A, Semenova A, Garcia-Vazquez E. eDNA and specific primers for early detection of invasive species—A case study on the bivalve Rangia cuneata, currently spreading in Europe. Mar Environ Res. 2015;112: 48–55. 10.1016/j.marenvres.2015.09.013 26453004

[42] Lacoursière-Roussel A, Côté G, Leclerc V, Bernatchez L. Quantifying relative fish abundance with eDNA: A promising tool for fisheries management. J Appl Ecol. 2016; 10.1111/1365-2664.12598

[43] Valentini A, Taberlet P, Miaud C, Civade R, Herder J, Thomsen PF, et al Next-generation monitoring of aquatic biodiversity using environmental DNA metabarcoding. Mol Ecol. 2016;25: 929–942. 10.1111/mec.13428 26479867

[44] Zaiko A, Martinez JL, Ardura A, Clusa L, Borrell YJ, Samuiloviene A, et al Detecting nuisance species using NGST: Methodology shortcomings and possible application in ballast water monitoring. Mar Environ Res. Elsevier Ltd; 2015;112: 64–72. 10.1016/j.marenvres.2015.07.002 26174116

[45] Hajibabaei M, Spall JL, Shokralla S, Van Konynenburg S. Assessing biodiversity of a freshwater benthic macroinvertebrate community through non- destructive environmental barcoding of DNA from preservative ethanol. BMC Ecol. 2012;12: 1. 23259585 10.1186/1472-6785-12-28 PMC3542036

[46] Ardura A, Planes S, Garcia-Vazquez E. DNA barcoding of fish landings Applications of DNA barcoding to fish landings: authentication and diversity assessment. 2013. 10.3897/zookeys.365.6409 PMC3890670 24453550

[47] Johnson R. Standardisation of river classifications. Sustain Dev. 2001;4: 11 Available: http://scholar.google.com/scholar?hl=en&btnG=Search&q=intitle:Standardisation+of+river+classifications+:#6

[48] Hebert PDN, Cywinska A, Ball SL, Jeremy R. Biological identifications through DNA barcodes. Proc R Soc Lond B. 2003;270: 313–321. 10.1098/rspb.2002.2218 12614582 PMC1691236

[49] Briski Ghabooli Sara Bailey Sarah MacIsaac EA Hugh J. Are genetic databases sufficiently populated to detect non-indigenous species? Biol Invasions. 18 10.1007/s10530-016-1134-1 PMC7175672 32355454

[50] Ardura A, Morote E, Kochzius M, Garcia-Vazquez E. Diversity of planktonic fish larvae along a latitudinal gradient in the Eastern Atlantic Ocean estimated through DNA barcodes. PeerJ. PeerJ Inc.; 2016;4: e2438 10.7717/peerj.2438 27761307 PMC5068340

[51] Taberlet P, Coissac E, Pompanon F, Brochmann C, Willerslev E. Towards next-generation biodiversity assessment using DNA metabarcoding. Mol Ecol. 2012;21: 2045–2050. 10.1111/j.1365-294X.2012.05470.x 22486824

[52] Rees HC, Maddison BC, Middleditch DJ, Patmore JRM, Gough KC. REVIEW: The detection of aquatic animal species using environmental DNA–a review of eDNA as a survey tool in ecology. J Appl Ecol. 2014;51: 1450–1459.

[53] Bohmann K, Evans A, Gilbert MTP, Carvalho GR, Creer S, Knapp M, et al Environmental DNA for wildlife biology and biodiversity monitoring. Trends Ecol Evol. 2014;29: 358–367. 10.1016/j.tree.2014.04.003 24821515

[54] Nislow KH, Hudy M, Letcher BH, Smith EP. Variation in local abundance and species richness of stream fishes in relation to dispersal barriers: Implications for management and conservation. Freshw Biol. 2011;56: 2135–2144. 10.1111/j.1365-2427.2011.02634.x

[55] Cooper AR, Infante DM, Wehrly KE, Wang L, Brenden TO. Identifying indicators and quantifying large-scale effects of dams on fishes. Ecol Indic. 2016;61: 646–657. 10.1016/j.ecolind.2015.10.016

[56] Santos RMB, Sanches Fernandes LF, Cortes RMV, Varandas SGP, Jesus JJB, Pacheco F a. L. Integrative assessment of river damming impacts on aquatic fauna in a Portuguese reservoir. Sci Total Environ. Elsevier B.V.; 2017;601–602: 1108–1118. 10.1016/j.scitotenv.2017.05.255 28599367

[57] Hodgson JA, Thomas CD, Wintle BA, Moilanen A. Climate change, connectivity and conservation decision making: back to basics. J Appl Ecol. Wiley/Blackwell; 2009;46: 964–969. 10.1111/j.1365-2664.2009.01695.x

[58] Elbrecht V, Vamos EE, Meissner K, Aroviita J, Leese F. Assessing strengths and weaknesses of DNA metabarcoding-based macroinvertebrate identification for routine stream monitoring. Methods Ecol Evol. 2017;8: 1265–1275. 10.1111/2041-210X.12789

[59] Sigsgaard EE, Carl H, Møller PR, Thomsen PF. Monitoring the near-extinct European weather loach in Denmark based on environmental DNA from water samples. Biol Conserv. Elsevier Ltd; 2015;183: 46–52. 10.1016/j.biocon.2014.11.023

